# In Situ Fabricated Liquid Metal Capacitors for Plant Sensing

**DOI:** 10.3390/bios13060603

**Published:** 2023-06-01

**Authors:** Sen Chen, Muzhi Jiang, Bo Wang, Xiyu Zhu, Xiaohui Shan, Jing Liu

**Affiliations:** 1 Department of Biomedical Engineering, School of Medicine, Tsinghua University, Beijing 100084, China; 2 Beijing Key Lab of Cryo-Biomedical Engineering and Key Lab of Cryogenics, Technical Institute of Physics and Chemistry, Chinese Academy of Sciences, Beijing 100190, China; 3 School of Biological Science and Medical Engineering, Beihang University, Beijing 100083, China

**Keywords:** liquid metal, capacitive sensors, plant sensing, water monitoring

## Abstract

Capacitive sensors are essential to promoting modernization and intelligence in agriculture. With the continuous advancement of this sensor technology, the demand for materials with high conductivity and flexibility is rapidly increasing. Herein, we introduce liquid metal as a solution for the in-site fabrication of high-performance capacitive sensors for plant sensing. As a comparison, three pathways have been proposed for the preparation of flexible capacitors inside plants, as well as on their surfaces. Specifically, concealed capacitors can be constructed by directly injecting liquid metal into the plant cavity. Printable capacitors are prepared via printing Cu-doped liquid metal with better adhesion on plant surfaces. A composite liquid metal-based capacitive sensor is achieved by printing liquid metal on the plant surface and injecting it into the interior of the plant. While each method has limitations, the composite liquid metal-based capacitive sensor provides an optimal trade-off between signal capture capability and operability. As a result, this composite capacitor is chosen as a sensor for monitoring water changes within plants and demonstrates the desired sensing performance, making it a promising technology for monitoring plant physiology.

## 1. Introduction

With the world’s population rapidly growing, food security has become one of the major challenges for humanity. In order to solve this problem, expanding the area under cultivation for food is a straightforward way of doing this, but it often results in the destruction of forests and grasslands, which is detrimental to environmental protection. Especially in the current situation of global warming and excessive CO_2_ emissions, expanding the area under food cultivation should be approached with caution. Therefore, increasing the productivity of food per unit area is another viable option. Precision agriculture, a modern agricultural production system that utilizes information and knowledge management, is dedicated to applying information technology to the whole process of crop production to achieve maximum agricultural productivity and is an effective way to achieve sustainable agriculture with high quality, high yield, low consumption, and environmental protection. Therefore, precise agriculture has gained considerable attention in recent years [1,2,3].

To achieve precision agriculture, various sensing technologies are being used for plant sensing and monitoring. Resistive and capacitive sensors are used to acquire data during plant growth [4,5,6]. Compared with resistive sensors, capacitive sensors have the advantages of fast corresponding speed, high sensitivity, and easy operation [7,8]. Moreover, in many cases, the resistance of plants varies weakly, which is not conducive to reflecting the change in the signal to be measured, thus limiting the application and expansion of resistance sensors in the field of plant monitoring. As a result, capacitive sensors are becoming increasingly useful to test the physiological status of plants. However, because of the naturally existing rough structure on the plant surface, it is difficult for the commonly used capacitive sensors to be completely patched to the plant surface, which is not conducive to accurate measurement. Thus, it is crucial to choose the right material for the capacitive sensors.

To address this challenge, liquid metal was recently selected as a plant sensing material for its excellent biocompatibility [9,10], high conductivity [11,12], and good liquidity [13,14]. For instance, Wu’s team printed liquid metal onto the surface of plants, enabling monitoring of the plant growth process [6,15]. To find out further generalized ways towards tackling the challenges facing the area, we proposed the basic principle of liquid metal plant injectable electronics for addressing various physiological interactions [15]. Using its inherent low viscosity, the liquid metal can be delivered directly into the plant cavity through a syringe, thus enabling completely concealed electronics, as desired. Along this way, some sensing elements, such as capacitors, were developed. However, the original capacitors implemented there have certain limitations due to a single structure. Considering such sensing has a unique value in plant electronics, here we further combined the printing strategies with the injection together and constructed a class of composite liquid–metal-based capacitive sensors. To demonstrate the advantages of this composite sensor, other liquid metal capacitors with different geometrical configurations were assessed and compared. A series of plant experiments confirmed that this composite capacitive sensor can well capture the capacitance changes during the loss of water, proving its application value in monitoring physiological changes in plants.

## 2. Materials and Methods

The water spinach used in our experiments was purchased from an agricultural market and grown in nutrient soil under natural light at room temperature. The plants were watered once a week and received fertilizer when they were first planted. Bamboo comes from gardens on campus. The liquid metal used for injection was EGaInSn, which consists of 68 wt% gallium, 22 wt% In, and 10 wt% Sn. EGaInSn is made by adding a certain proportion of weighed metal components into the glass and heating it to 200 °C for about an hour until all the metals are melted into liquid. After the liquid metal was injected, a wire was just inserted into the needle hole, and then it was fixed by silicone rubber. The other hole for degassing was also sealed by silicone rubber. It took 24 h for the silicone rubber to become totally solid. To achieve the printing of EGaInSn, 1 μm Cu nanoparticles were added into EGaInSn via stirring EGaInSn and Cu nanoparticles for 30 min in air. The instrument used is a mechanical stirrer with a speed of 600 r/min. To demonstrate that the liquid metal printed on the plant surface can serve as common patch electrodes, we first tried to fabricate it on Venus fly traps. To obtain a liquid metal printed electrode with a specific shape, we first designed the shape of printed electrodes in Solidworks, and the mold composed of PDMS was then obtained by 3D printing. The mass ratio of PDMS to curing agent is 10:1. The PDMS mask was then pasted to the fly trap, and the liquid metal was brushed onto the surface by line drawing pens. After that, a kind of silica gel line was brushed into the surface of liquid metal as an insulating layer. Finally, we measured the capacitance with a LCR digital bridge capacitance resistance measuring instrument (TH2832, Tonghui, China). The frequency chosen for the water content test experiment was 1 kHz.

## 3. Results

**Figure 1:**
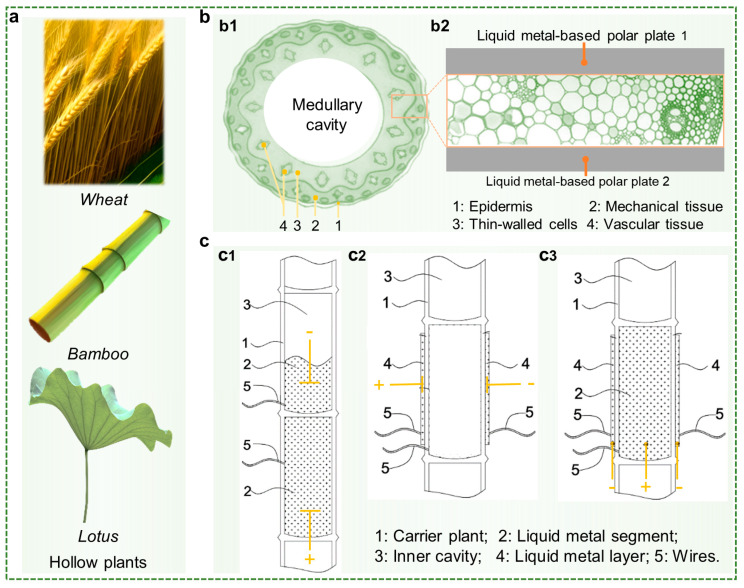
Three solutions for liquid metal-based capacitive sensors. (**a**) Typical plants with hollow structures. (**b**) (**b1**) Schematic diagram of the structure of the hollow stem of a plant. (**b2**) Structure of a capacitive sensor based on the structure of a cavity inside a plant. (**c**) Different types of capacitive sensors, including injectable capacitors consisting of two sections of liquid metal injected into the inside of the plant (**c1**), printable capacitors consisting of liquid metal printed onto the surface of the plant (**c2**), and composite capacitors achieved by injecting liquid metal into the inside of the plant and printing liquid metal onto the surface of the plant (**c3**).

In previous studies, capacitive sensors were usually placed on the surface of the plant, which is easy to manipulate and controllable. In this study, we placed one or two electrodes of the capacitive sensor inside the plant by injection, based on the principle of plant injectable electronics [16]. To achieve successful injection of foreign substances while minimizing damage to the plant by injection, naturally existing cavities within the plant are utilized. As illustrated in Figure 1a, plants possess a variety of categories, some of which evolved to acquire cavity structures, and wheat, bamboo, and lotus are typical examples. A cross-section of the internal hollow structure of a plant is shown in Figure 1b. The cavity is usually formed because the pith of the stem of these plants shrinks and disappears during growth, thus providing nutrients for the construction of stronger mechanical tissue and vascular tissues. Utilizing these cavities, an envisioned capacitance sensor can be obtained, where the electrode plates consist of liquid metal placed in the internal cavity or the surface of the plants. Given that the plant tissue is between the two liquid metal polar plates, the change in the value measured by the capacitance sensor thus constructed will be used to reflect the variation in the plant tissue.

Three types of capacitive sensors can be obtained, depending on the position in which the liquid metal electrode plate is located (Figure 1c). The electrode plates of the first type of capacitor are located in two connected cavities inside the plant, with the plant tissue separating the cavities in between (Figure 1(c1)). Considering that all of its electrode plates can be obtained by injection, it is referred to in this study as an injectable capacitor. The liquid metal electrode plates in the second type of capacitor are located on both surfaces of the plant, as shown schematically in Figure 1(c2), which is called as printable capacitor. The third type of composite capacitor makes use of both the cavity inside the plant and the surface of the plant, with electrode plates composed of liquid metal placed in them individually, as shown in Figure 1(c3). Each of these three types of capacitive sensors has its own characteristics, which will be demonstrated in the subsequent study. Moreover, we will also select suitable capacitive sensors for application presentation.

**Figure 2:**
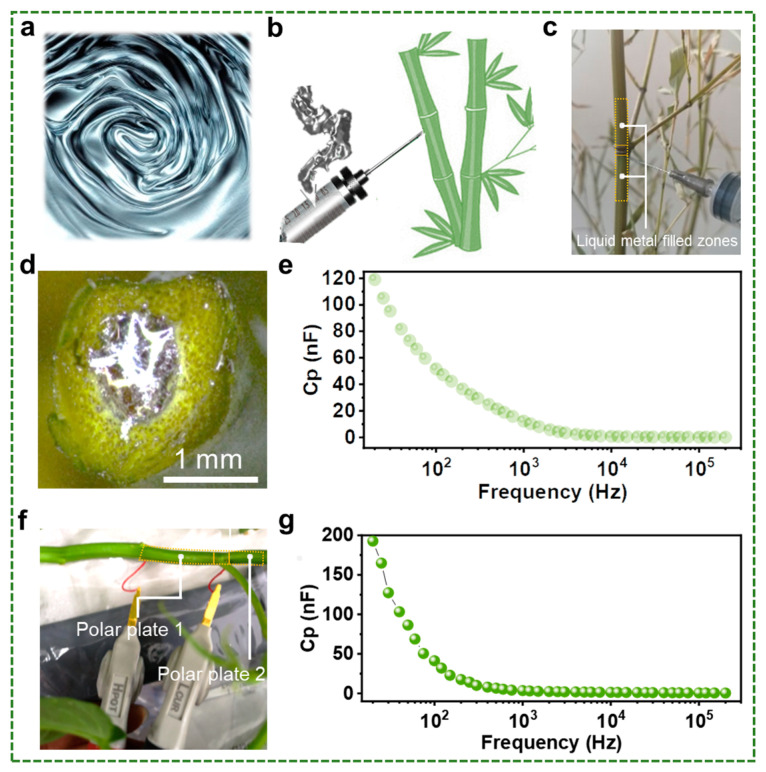
Preparation method and test results of injectable liquid metal capacitors. (**a**) Liquid metal with good flowability. (**b**) Schematic diagram of injection of liquid metal into a plant. (**c**) Injecting liquid metal into the sides of the bamboo between the nodes. (**d**) Liquid metal inside the plant cavity. (**e**) Plot of capacitance value (*Cp*) versus frequency for a liquid metal capacitance sensor constructed inside a bamboo. (**f**) Injectable liquid metal capacitor constructed inside water spinach. (**g**) Variation in the capacitance value (*Cp*) of the capacitance sensor inside the cavity of plants with frequency.

To achieve the injectable capacitor within the plant, liquid metal needs to be injected into the cavity of the plant. Fortunately, the liquid metal used in this study is a eutectic gallium-indium-tin alloy (EGaInSn), which has about twice the viscosity of water [17] and exhibits excellent injectable properties (Figure 2a). Besides, the liquid metal possesses a melting point of 10.5 °C and a relatively large stable undercooling, which means that the liquid metal is liquid in most use cases, providing a wide range of injectable scenarios. Specifically for experimental operations, syringes are used to inject liquid metal (Figure 2b), which is simple and allows for quantitative injection. For hard plants, such as bamboo, it is necessary to drill an injection port with a suitable hole diameter in advance before injection. To monitor the tissues within the bamboo, liquid metal should be injected into both sides of the bamboo internodes, as illustrated in Figure 2c. To determine that the liquid metal can adhere well to the plant, we observed the profile of the liquid metal inside the plant. As illustrated in Figure 2d, the liquid metal is in good contact with the plant tissue. The reason behind this is that the large surface tension of liquid metals is not conducive to their spreading, yet the oxides easily generated on their surfaces allow them to be adhered to plant surfaces [18].

The study further tested the performance of the capacitor inside the bamboo. As illustrated in Figure 2e, the capacitor performance decreases constantly with increasing test frequencies. For the actual test, the real circuit model is complicated. The AC feature of the capacitor could be affected by the parasitic inductance and parasitic resistance, and the apparent capacitance (*C_a_*) is reflected by the reactance of the whole equivalent circuits. To better illustrate this issue, the circuit model can be simplified, which is depicted by the following formula:

(1) $$C_{a}=\frac{1}{2\pi fX_{C}}$$

where; $X_{C}$ is capacitive reactance, and *f* is frequency. Hence, with the current frequency going up, the tested capacitance should go down, which is in agreement with the experimental results. Besides, the measuring error of the capacitance can be regarded as spurious impedance illustrated in series or parallel. Depending on the level of spurious impedance, different test modes are selected. When the impedance is large, the parallel equivalent capacitance (*Cp*) is suitable, while, when the impedance is small, the series equivalent capacitance (*C_S_*) should be preferred. Here, given the relatively high impedance of the plant tissue, *Cp* was chosen to reflect the characteristics of capacitors, which can be used to reflect the variation in plant growth conditions. As a comparison, water spinach was also selected. Figure 2f illustrates the test procedure of the capacitance inside the water spinach, where the liquid metal inside the stem is not visible from the outside. The results of the test are also shown in Figure 2g. Compared to bamboo, the value of the capacitor formed by the injection of liquid metal inside the water spinach is larger at the initial stage of the test (quite low frequency range). We speculate that the mechanism lying behind this difference is related to the water content of the plant, which can be understood because bamboo stems are woody and generally contain less water than the soft stems of water spinach. Certainly, the factors affecting the capacitance of different plants are closely related to their own tissue properties in addition to moisture. The difference in capacitance values of liquid metal in bamboo and water spinach also indicates the feasibility of liquid metal-based capacitance sensors prepared based on this injectable method.

**Figure 3:**
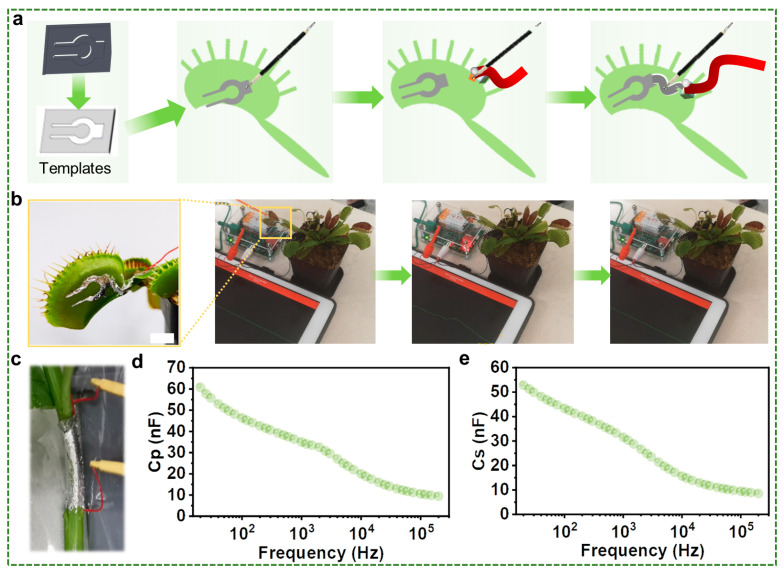
Preparation process and performance testing of liquid metal printable capacitors. (**a**) Preparation process of printed electrodes based on liquid metal. (**b**) Liquid metal electrodes on flycatcher. (**c**) Liquid metal printable capacitor on the surface of water spinach. (**d**) Parallel equivalent capacitance (*Cp*) of liquid metal printable capacitors on the surface of water spinach. (**e**) Series equivalent capacitance (*C_S_*) of liquid metal printable capacitors on the surface of water spinach.

In addition to injectable liquid metal capacitors within plants, printable liquid metal capacitors have also been prepared on the surface of the plant. The success of this fabrication method relies heavily on the effectiveness of the liquid metal printable electrodes in capturing signals from the plants. To ensure this, we conducted an experiment using a flycatcher as a model organism. The liquid metal was first printed onto the surface of the flycatcher, and then the plant was stimulated to observe any changes. The goal was to test whether the printed electrodes could effectively capture this typical biological event. Figure 3a illustrates the preparation process of liquid metal printable electrodes. To achieve a better printable effect, copper particles were added into the liquid metal to reduce its surface tension, allowing it to be successfully printed onto the epidermis of plants. Moreover, the highly conductive copper particles could partially offset the loss of electrical conductivity of the liquid metal caused by oxidation. Further, we tested the active potential by touching the small villi in the fly traps, and it is found that it just worked as well as the traditional electrode (Figure 3b).

Figure 3c illustrates the printing of liquid metal into the surface of water spinach, an experiment whose results are depicted in Figure 3d,e. The data reveal that the value of capacitance in the printable liquid metal capacitive sensors is significantly smaller than that performed through injecting. The reason for this disparity is that the plant used in the experiment contains internal cavities that increase the distance between the two electrode plates and alter the dielectric constant, leading to a substantial reduction in the capacitance. To elaborate further, the presence of internal cavities in the plant leads to an increase in the distance between the two electrode plates, reducing the capacitance of the sensors. Additionally, the cavities alter the dielectric constant, further contributing to the decline in capacitance. These changes in capacitance values can be used to detect variations in environmental factors, such as humidity or temperature. However, the resulting liquid metal capacitive sensors are not very robust and are susceptible to external influences such as rainstorms, which can damage the delicate sensors and compromise their functionality.

**Figure 4:**
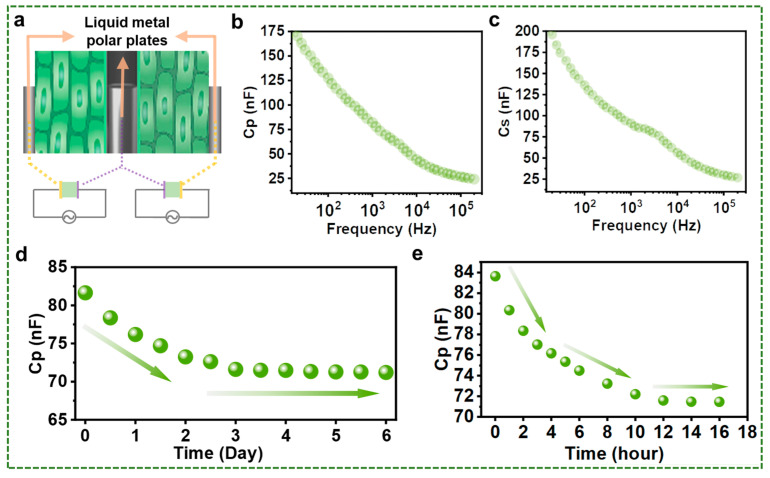
Composite capacitors based on liquid metal injection and printing and their applications. (**a**) Schematic diagram of a composite capacitor. (**b**) Parallel equivalent capacitance (*Cp*) of liquid metal composite capacitors. (**c**) Series equivalent capacitance (*C_S_*) of liquid metal composite capacitors. (**d**) The trend in liquid metal composite capacitance during water loss in bamboo. (**e**) The trend of the capacitance value of liquid metal obtained by accelerating the evaporation of water during heating.

Further research is necessary to enhance the durability of these sensors and minimize their vulnerability to external factors. Therefore, combining the advantages of the above two strategies, here we propose a liquid metal-based composite capacitive sensors via printing liquid metal on the surface of plants and injecting liquid metal into the cavity of plants. The structure diagram of such composite capacitors is shown in Figure 4a. Figure 4b,c show the capacitance value test results of this capacitor, showing that the capacitor constructed by this method can achieve the capacitance function very well. For the same part of the same plant, the capacitance value obtained via this method is larger than the capacitance value measured by the printing methods. The reason behind this is that the pole plate distances of the capacitors prepared by these two strategies are inconsistent. The distance between the two pole plates forming the composite capacitor is smaller than that of the capacitive sensor formed by direct printing. The resulting composite capacitor obtains significant capacitance values while requiring only one electrode plate to be located externally, thereby greatly reducing the influence of external factors on the electrode plate compared to a printed capacitor. In addition, multiple capacitive sensors can share a single injectable electrode plate to achieve sensing of target plant tissues, which greatly increases operability compared to an injectable capacitor.

As a demonstration of the application, such a composite capacitor is used to test the water content inside the plant. Water is an indispensable substance for plant growth and plays a highly important role in the physiological health of plants [19,20]. Besides, it is known that the dielectric constant of water is about 80 [21], which is significantly larger than the common gas and organic materials. Thus, the water content in the material has a noticeable effect on the capacitance value of the capacitor, which is illustrated by a series of studies [22,23,24]. For example, the moisture content of plants can be obtained via such sensing technology [25,26,27]. In addition, the calculation formula for the capacitance value of a cylindrical capacitor is shown below:

(2) $$C=\frac{2\pi \varepsilon_{0}L}{\ln\left(\frac{R}{r}\right)}$$

where; $\varepsilon_{0}$ is permittivity, *L* is the height of the cylinder, *R* is the exradius, and *r* is the inner radius. It can be seen that the change in capacitor size and the variations in the dielectric constant of the inter-pole dielectric affect the capacitance value. Additionally, it is known from the previous discussion that the dielectric constant variation of plant tissues is mainly related to the water content. Therefore, the change in the value of the plant-related capacitor can be a good indicator of the water changes within the plant.

To ensure that the water content is represented as much as possible by the capacitance value of the capacitor, bamboo with very little change in shape during the water loss process was selected. Figure 4d shows the results of the experiment, and it is clear from the figure that the capacitance value of the capacitor shows a decreasing trend in general. Moreover, the changes in capacitance values are mainly concentrated in the first phase of the experiment. To better characterize this stage, we further tested the capacitance change in the bamboo in the heated state (35 °C), as shown in Figure 4e. Compared to the previous experimental results, the results of this experiment show that the value of capacitance decreases more rapidly in the initial phase, which in turn confirms the rapid loss of water. Water content experiments have numerous practical applications across various fields, such as agriculture, construction, geology, and environmental studies. These experiments can provide crucial insights into the water content of plant tissue. Although the primary purpose of the experiment may be to introduce a new concept, it also offers valuable information for the water content testing process. While there are many factors to consider when testing for water content, this study demonstrates that the proposed strategy is not only feasible, but also effective. This suggests that further research and exploration in this direction is highly encouraged, as it has the potential to contribute significantly to detecting the change in\ water content and its applications.

## 4. Conclusions

In summary, we have successfully constructed three types of liquid metal capacitive sensors, both on the surface and inside of plants, by printing and injection. Injectable or transplanted capacitors are realized by injecting liquid metal into the plant’s interior, which effectively characterizes and monitors the tissue situations inside the plant. While the capacitance values measured by liquid metal capacitors printed on the surface of liquid metal are significantly smaller than those measured by injectable capacitors, they are superior in their ease of handling, controllability, and ability to effectively respond to plant-specific tissue conditions. By combining the above two strategies, we injected and printed liquid metal into the interior and surface of the plant, respectively, thus realizing a composite capacitive sensor. This capacitor displays a larger capacitance value than the printed capacitor, while its maneuverability is greatly increased by the need for only one pole plate to be injected into the plant. Furthermore, based on this obtained composite capacitive sensor, we successfully monitored the water loss process of bamboo, demonstrating its potential application value. In conclusion, it is believed that liquid metal-based capacitive sensors hold significant promise in the field of plant sensing and monitoring.

## Data Availability

Data are contained within the article.
